# Establishment of a dual droplet digital PCR method for detecting the *Brucella abortus* A19-ΔVirB12 strains

**DOI:** 10.3389/fmicb.2025.1684156

**Published:** 2025-10-01

**Authors:** Ruixue Xue, Zunfeng Chu, Linlin Xing, Zixin Jiang, Wenduo Jiang, Yingli Shang, Fangkun Wang, Hongmei Wang, Yuyu Zhang, Yanling Wang, Yu Miao, Xinglin Zhang, Mingjun Sun, Zouran Lan, Yue Zhang

**Affiliations:** ^1^Shandong Provincial Center for Animal Disease Prevention and Control (Shandong Provincial Center for Zoonoses Epidemiology Investigation and Surveillance), Jinan, Shandong, China; ^2^Shandong Provincial Key Laboratory of Zoonoses Diseases, Jinan, Shandong, China; ^3^Department of Preventive Veterinary Medicine, College of Veterinary Medicine, Shandong Agricultural University, Taian, Shandong, China; ^4^Ruminant Diseases Research Center, College of Life Sciences, Shandong Normal University, Jinan, Shandong, China; ^5^Key Laboratory of Livestock and Poultry Multi-Omics of MARA, Institute of Animal Science and Veterinary Medicine, Shandong Academy of Agricultural Sciences, Jinan, Shandong, China; ^6^College of Veterinary Medicine, Henan Agricultural University, Zhengzhou, Henan, China; ^7^Shandong Sinder Technology Co., Ltd., Weifang, Shandong, China; ^8^China Animal Health and Epidemiology Center, Qingdao, Shandong, China

**Keywords:** brucellosis, A19-ΔVirB12 strain, *VirB8* gene, *VirB12* gene, ddPCR

## Abstract

Brucellosis is a zoonosis that occurs worldwide, and vaccination is the main strategy for controlling it. In China, the *Brucella abortus* A19-ΔVirB12 strain is utilized in main vaccines. However, a high-sensitivity nucleic acid detection method to effectively differentiate *Brucella* infections from immunization with the A19-ΔVirB12 strain is lacking. Therefore, in this study, a duplex droplet digital PCR (ddPCR) assay was established using primers and probes targeting the *VirB8* gene and the deleted *VirB12* gene in the A19-ΔVirB12 strain. The specificity of the method was tested using genomic DNA of *Mycobacterium bovis*, *Escherichia coli* (O:157), *Salmonella* spp., *Streptococcus* spp., and A19-ΔVirB12 *Brucella*. Only A19-ΔVirB12 amplified *VirB8* gene. The detection limits of the method for *VirB8* and *VirB12* were 2.13 × 10^0^ and 2.26 × 10^0^ copies/μL, respectively. In the detection of DNA in epidemic-related samples, the positive rate of ddPCR was much higher than that in the samples analyzed using the commercial fluorescence quantitative reagent kits. Meanwhile, the ddPCR of the A19-ΔVirB12 *Brucella* vaccine strain was identified in the clinical samples. In summary, the ddPCR method with high sensitivity and specificity was established, which will support the future identification of A19-ΔVirB12 *Brucella* vaccine strains in immunized and wild-type *Brucella*.

## 1 Introduction

Brucellosis is a zoonotic disease that impacts livestock and humans worldwide, leading to chronic conditions such as undulant fever, weakness, myalgia, and arthralgia (Franco et al., 2007; Dean et al., 2012; Esmaeilnejad-Ganji and Esmaeilnejad-Ganji, 2019). Humans contract the disease through different routes, including the ingestion of unpasteurized milk and dairy products; direct contact with infected animal tissues; or accidental ingestion, inhalation, or injection of cultured *Brucella* (Young, 1983; Corbel et al., 2006; Dadar et al., 2019). More than 500,000 human cases are reported annually worldwide (Pappas et al., 2006; Seleem et al., 2010), although the true number of cases is likely to be much higher due to inaccurate diagnosis, inadequate surveillance, and incomplete reporting (Di Bari et al., 2022; Laine et al., 2023).

Managing brucellosis in livestock is a necessary and cost-effective way to decrease the number of human cases of brucellosis (Golshani and Buozari, 2017). Accurate diagnosis of this bacterial disease is a fundamental challenge, as is finding a suitable medication to eradicate it (Helmy et al., 2025). In China, the main control strategy for brucellosis outbreaks involves vaccination along with quarantine and culling. The *Brucella abortus* A19 vaccine strain is the most effective and widely used for cattle immunization (He et al., 2022). However, the antibody response induced by the O-side chain of the A19 vaccine interferes with serological diagnosis, making it difficult to distinguish between vaccinated and infected animals (Perkins et al., 2010). Therefore, the development and application of gene-deleted vaccines is needed to overcome this limitation (Weiss et al., 2015; Lin et al., 2020).

The A19-ΔVirB12 gene-deleted vaccine, which carries a diagnostic marker, exhibits a protective efficacy comparable to that of the parental A19 strain but demonstrates lower virulence (Yang et al., 2021). The vaccine strain, which can be distinguished from naturally infected strains using *VirB12* gene differential diagnostic methods, was officially launched in 2021 in China. However, no a high-sensitivity nucleic acid detection method for nucleic acid-based differentiation exists.

Droplet digital PCR (ddPCR) is a highly precise nucleic acid quantification technology, with greater sensitivity and absolute quantitative capabilities compared to conventional quantitative PCR (qPCR) (Hindson et al., 2011; Quan et al., 2018; Sancha Dominguez et al., 2024). In this study, primers targeting the *VirB8* gene of the type IV secretion system were designed for the genus-level identification of *Brucella*. The *VirB12* gene is deleted in the A19-ΔVirB12 strain and serves as a diagnostic marker. The objective of this study was to establish a duplex ddPCR assay to differentiate between the A19-ΔVirB12 vaccine strain from field strains of *Brucella abortus*, providing a rapid and reliable diagnostic method for distinguishing vaccinated cattle from those infected with wild-type *Brucella*. A duplex ddPCR method for identifying A19-ΔVirB12 strain was successfully developed by optimizing parameters, such as primer concentration, probe concentration, and annealing rate, along with the evaluation of sensitivity, specificity and repeatability. This method was successfully applied to both vaccine samples and clinical specimens, indicating its potential a novel and effective technical approach for early detection, accurate diagnosis and scientific control of brucellosis.

## 2 Materials and methods

### 2.1 Vaccines and samples

A19-ΔVirB12 *Brucella* vaccine was purchased from Tecon Biology Co., Ltd. Additionally, 53 clinical samples were collected from some areas’ cattle farms with symptoms of miscarriage from various cattle farms in the cities of Yantai, Zibo, Weifang, Liaocheng, Weihai, and Dongying in the Shandong Province to evaluate the practical performance of the ddPCR assay.

Nucleic acids of *Mycobacterium bovis*, *Escherichia coli* (O:157), *Salmonella* spp., *Streptococcus* spp. and *Bacillus anthracis*, preserved at the Shandong Animal Disease Prevention and Control Center, were used to assess assay specificity.

### 2.2 DNA extraction

Milk samples: 200 μL of milk was taken into a 1.5 mL centrifuge tube containing PBS. The samples were centrifuged at 12,000 × g for 5 min, the supernatant was discarded, and residual milk fat was wiped off with a sterile cotton swab. DNA was extracted from these above samples using a Bacteria DNA Extraction Kit (Vazyme Biotech, Nanjing, China).

Vaginal swabs: the swab heads were immersed in 1 mL of PBS and mixed with shaking. The PBS soaked in the swab was transferred to a 1.5 mL centrifuge tube and centrifuged at 12,000 × g for 5 min, and the upper layer of liquid was discarded. DNA was extracted from these above samples using a Bacteria DNA Extraction Kit (Vazyme Biotech, Nanjing, China).

### 2.3 Primer and probe design

**Figure 1:**
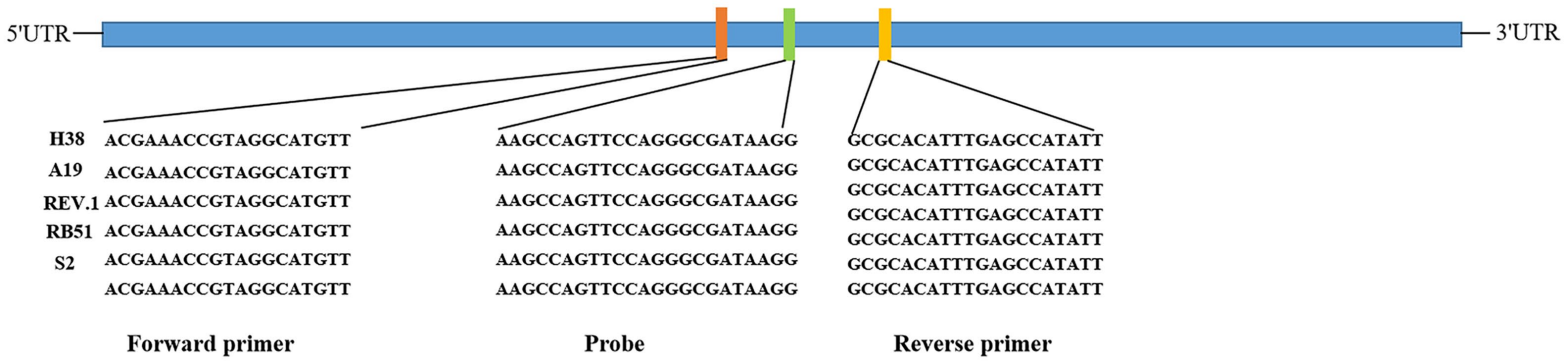
Result of *VirB8* gene sequence analysis.

The *VirB8* and *VirB12* genes sequences were obtained from GenBank (GenBank IDs: CP030752.1 and LT671513.1, respectively). After the consensus sequence was selected, primers and probes were designed using the DNAMAN software (Table 1). Sequence analysis conformed that the primers and probe were situated within highly conserved regions of the *VirB8* gene, as illustrated in Figure 1.

**Table 1:** Sequences of primers.

Primer name	Primer sequence	Amplified fragment length
*VirB8*-F	5′-ACGAAACCGTAGGCATGTT-3′	104 bp
*VirB8*-R	5′-GCGCACATTTGAGCCATATT-3′	
*VirB8*-Probe	5′-FAM-AAGCCAGTTCCAGGGCGATAAGG-BHQ1-3′	
*VirB12*-F	5′-GCCTGACGGACAACAACT-3′	99 bp
*VirB12*-R	5′-CGGGTACGCCTTGATTGAT-3′	
*VirB12*-Probe	5′-VIC-CGTGCGCTGGCTATCTATAATTGGCT-BHQ1-3′	

### 2.4 Construction of standard plasmids

*Brucella VirB8* and *VirB12* genes were amplified via primer pairs (Table 1). The reactions were carried out using 2 × Taq Master Mix (Vazyme Biotech, Nanjing, China). The PCR products were collected and purified using an EasyPure PCR Purification Kit (TransGen Biotech, Beijing, China), and the target sequences were cloned and inserted into the Trans1-T1 vector (TransGen Biotech, Beijing, China). The recombinant plasmids were subsequently purified with a FastPure EndoFree Plasmid Mini Plus Kit (Vazyme Biotech, Nanjing, China). The concentration of the plasmid DNA was measured with a NanoDrop 2000 spectrophotometer (Thermo Fisher Scientific, Delaware, United States). The initial concentrations of the *VirB8* and *VirB12* plasmids were 2.13 × 10^9^ copies/μL and 2.26 × 10^9^ copies/μL, respectively.

### 2.5 ddPCR reaction conditions

For the ddPCR reactions, the 30 μL reaction mixture contained 15 μL of ddPCR SuperMix (2×) (TargetingOne Biotech, Beijing, China), 1 μL of DNA template, forward and reverse primers, and probes (which were later optimized). The volume was adjusted to 30 μL with sterile deionized water. Subsequently, 30 μL of reaction mixture and 150 μL of droplet generation oil were loaded into a droplet generator cartridge, sealed with a gasket, and placed into the droplet generator to produce droplets. The generated droplets were transferred to a PCR thermocycler. Following ddPCR amplification, eight-strip tubes were placed in a droplet reader (Targetingone Biotech, Beijing, China) to analyze fluorescence signals.

### 2.6 Evaluation of the sensitivity of ddPCR

*VirB8* and *VirB12* standard plasmids were 10-fold serial gradient diluted with ddH_2_O, respectively. Serial dilutions were prepared as follows: *VirB8* plasmids, 2.13 × 10^8^–2.13 × 10^0^ copies/μL and *VirB12* plasmids, 2.26 × 10^8^–2.26 × 10^0^ copies/μL. For each dilution, 2 μL of plasmid DNA was used as the template in the established assay system; blank controls and replicates were used to evaluate sensitivity. Standard curves were plotted to display the results.

### 2.7 Evaluation of the specificity of ddPCR

Different sources of DNA from *Mycobacterium bovis*, *Escherichia coli* (O:157), *Salmonella* spp., *Streptococcus* spp., *Bacillus anthracis*, *Clostridium perfringens*, and A19-ΔVirB12 *Brucella* vaccine were diluted to 1 ng/μL and then tested to evaluate the specificity of the ddPCR assay.

### 2.8 Evaluation of ddPCR with clinical samples

Overall, 53 clinical samples were collected from cattle with incidences of miscarriage from farms in the cities of Yantai, Linyi, Qingdao, Liaocheng, Weihai, Rizhao, and Dongying in the Shandong Province from 2023 to 2024. To compare the efficacy of our method, the commercial fluorescence quantitative reagent kits (Lijian Biotech, Qingdao, China) were used in parallel with ddPCR to test clinical samples.

## 3 Results

### 3.1 Optimization of ddPCR reaction conditions

**Figure 2:**
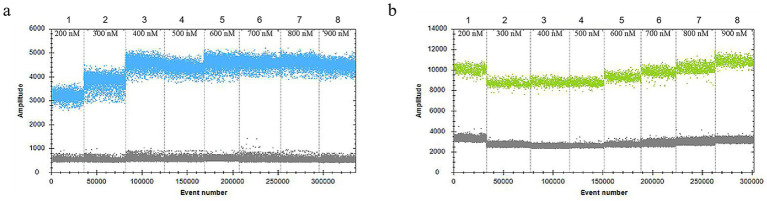
Optimization of primer concentrations of *VirB8* and *VirB12*. **(a)** Primer concentrations of *VirB*8; 1–8: 200 nM, 300 nM, 400 nM, 500 nM, 600 nM, 700 nM, 800 nM, and 900 nM, respectively. **(b)** Primer concentrations of *VirB12*; 1–8: 200 nM, 300 nM, 400 nM, 500 nM, 600 nM, 700 nM, 800 nM, and 900 nM, respectively.

**Figure 3:**
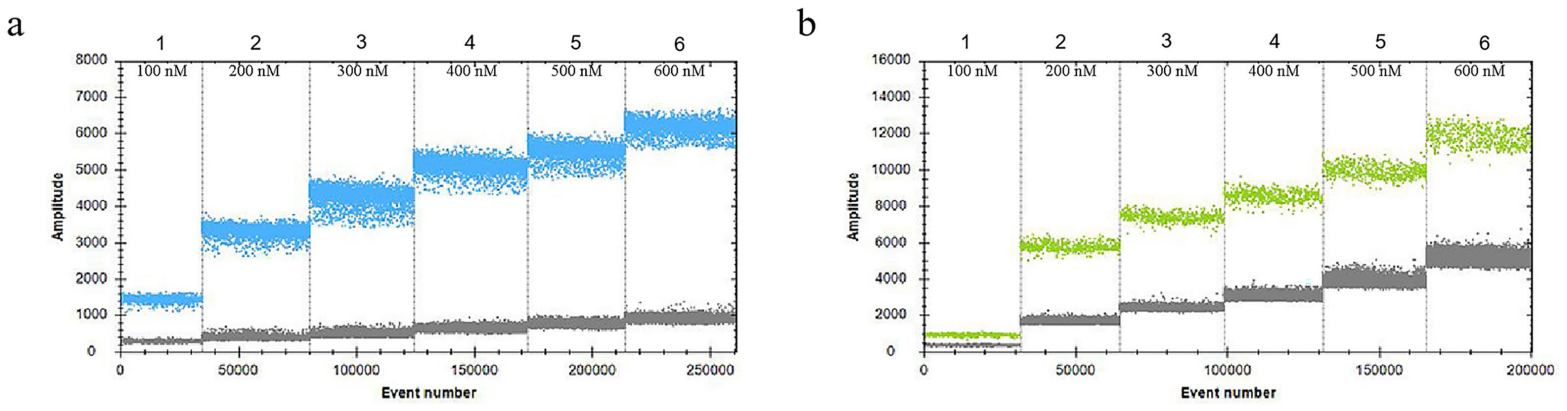
Optimization of probe concentrations of *VirB8* and *VirB12*. **(a)** Probe concentrations of *VirB8* in FAM channel; 1–6: 100 nM, 200 nM, 300 nM, 400 nM, 500 nM, and 600 nM, respectively. **(b)** Probe concentrations of *VirB12* in VIC channel; 1–6: 100 nM, 200 nM, 300 nM, 400 nM, 500 nM, and 600 nM, respectively.

**Figure 4:**
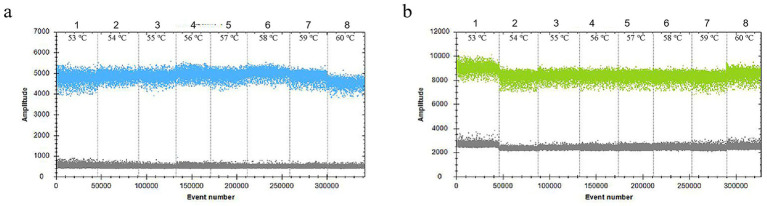
Optimization of the annealing temperature of ddPCR. **(a)** Annealing temperature of *VirB8* in FAM channel; 1–8: 53 °C, 54 °C, 55 °C, 56 °C, 57 °C, 58 °C, 59 °C, and 60 °C, respectively. **(b)** Annealing temperature of *VirB12* in VIC channel; 1–8: 53 °C, 54 °C, 55 °C, 56 °C, 57 °C, 58 °C, 59 °C, and 60 °C, respectively.

In the 30 μL reaction system, the volume of *VirB8* and *VirB12* probes (10 μmol/L) was fixed at 0.75 μL. Eight final primer concentrations for *VirB8* and *VirB12* (10 μmol/L) were tested at eight final concentrations: 200 nM, 300 nM, 400 nM, 500 nM, 600 nM, 700 nM, 800 nM, and 900 nM. All the primer concentrations effectively distinguished between positive and negative droplets for both *VirB8* and *VirB12*. A final primer concentration of 600 nM (equivalent to 1.8 μL of primer) was selected for *VirB8* and *VirB12* to optimize amplification efficiency and minimize “tailing” effects (Figure 2). With the primer volume fixed at 1.8 μL (10 μmol/L) in the 30 μL reaction system, six final probe concentrations for *VirB8* and *VirB12* (10 μmol/L) were evaluated at six final concentrations: 100 nM, 200 nM, 300 nM, 400 nM, 500 nM, and 600 nM. The results indicated that a probe concentration of 300 nM (equivalent to 0.9 μL of probe) provided the best balance between amplification efficiency, minimization of “tailing” and cost-effectiveness for both *VirB8* and *VirB12* (Figure 3). Subsequently, with fixed primer (1.8 μL) and probe (0.9 μL) volumes, the annealing temperature was optimized: 53 °C, 54 °C, 55 °C, 56 °C, 57 °C, 58 °C, 59 °C, and 60 °C. The separation between the positive and negative droplets was largest and clearest at 54 °C (Figure 4).

Considering the fluorescence signal intensity, stability, minimization of “tailing,” and cost-effectiveness, the optimized reaction mixture was established contained the following: 15 μL of ddPCR SuperMix (2×), 1 μL of DNA template, 1.8 μL of both forward and reverse primers for VirB8 and VirB12 (10 μmol/L), and 0.9 μL of each probe (10 μmol/L). The final reaction volume was adjusted to 30 μL with sterile deionized water. Optimized amplification conditions were as follows: initial denaturation at 95 °C for 10 min; 40 cycles of denaturation at 94 °C for 30 s and annealing at 54 °C for 1 min; followed by a final cooling step at 12 °C for 5 min. Under these conditions, fluorescence signals were concentrated, positive and negative droplets were clearly separated, no “tailing” was observed, and an optimal number of droplets and amplification efficiency were achieved.

### 3.2 Sensitivity of ddPCR

**Figure 5:**
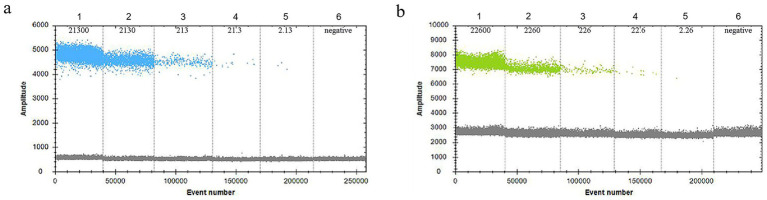
Sensitivity of ddPCR. **(a)** Sensitivity of *VirB8* in FAM channel; 1–5: 2.13 × 10^4^–2.13 × 10^0^ copies/μL dilution standards, 6: negative control. **(b)** Sensitivity of VirB12 in VIC channel; 1–5: 2.26 × 10^4^–2.26 × 10^0^ copies/μL dilution standards; 6: negative control.

Standard plasmids containing the *VirB8* and *VirB12* target sequences were serially diluted to nine concentrations and measured using the established duplex ddPCR assay. *VirB8* was consistently detected within the dilution range of 2.13 × 10^4^–2.13 × 10^0^ copies/μL, with a lower detection limit of 2.13 × 10^0^ copies/μL (Figure 5a). Similarly, *VirB12* was showed the detectable in the range of 2.26 × 10^4^–2.26 × 10^0^ copies/μL, with a lower detection limit of 2.26 × 10^0^ copies/μL (Figure 5b).

**Figure 6:**
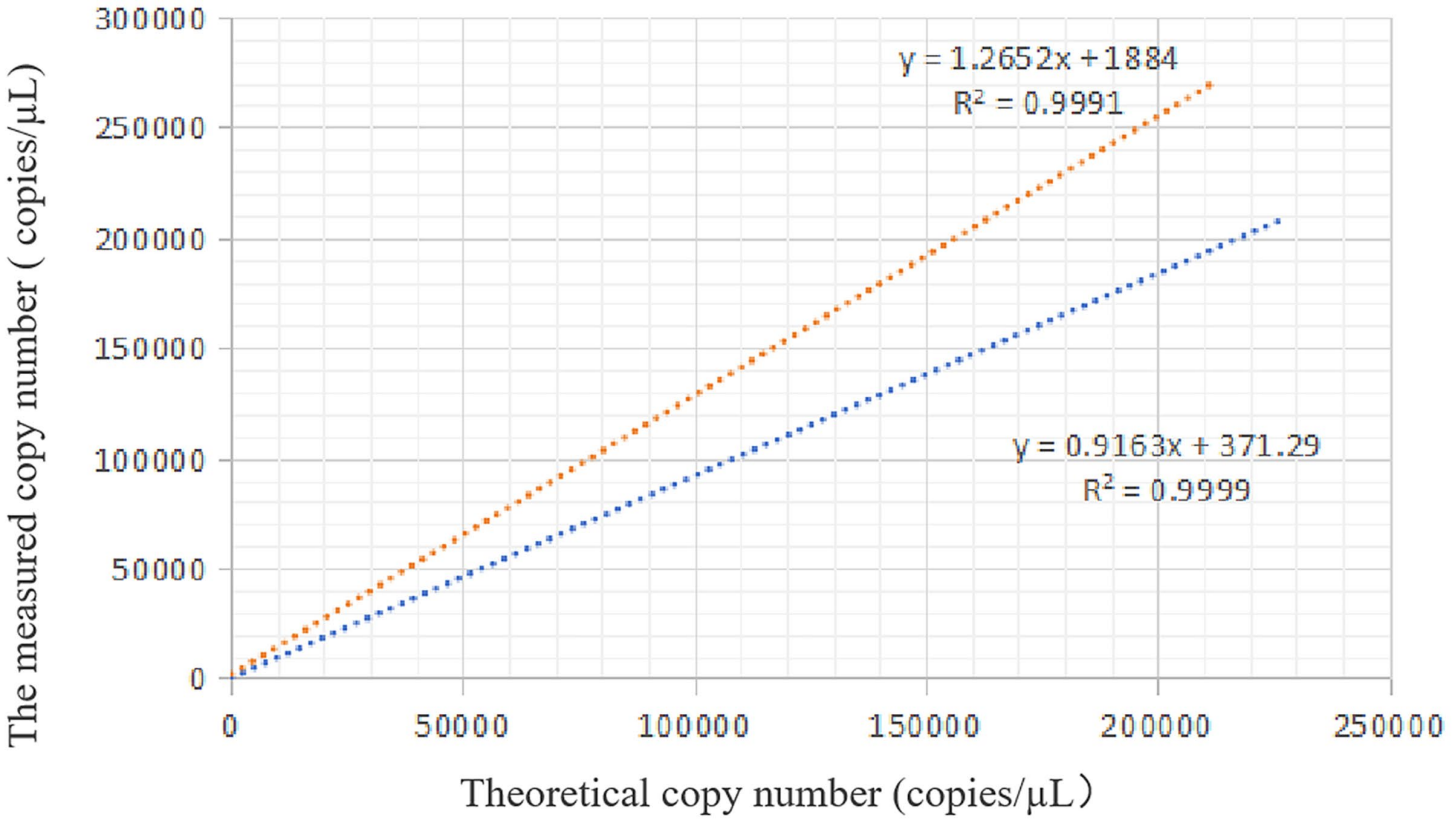
Standard curve of ddPCR. The orange dotted line is the linear regression analysis of *VirB8*, and the blue dotted line is the linear regression analysis of *VirB12*.

Based on the experimental results, the theoretical copy number was plotted on the x-axis and the measured copy number on the y-axis. For *VirB8*, the linear regression analysis yielded an *R*^2^ value of 0.9991 with the equation *y* = *1*.*2652x* + *1884*. For *VirB12*, the *R*^2^ value = 0.9999 with the equation *y* = *0*.*9163x* + *371*.*29*. Thus, the copy numbers determined by ddPCR were highly consistent with the theoretical values, demonstrating excellent linearity of the assay (Figure 6).

### 3.3 Specificity of ddPCR

**Figure 7:**
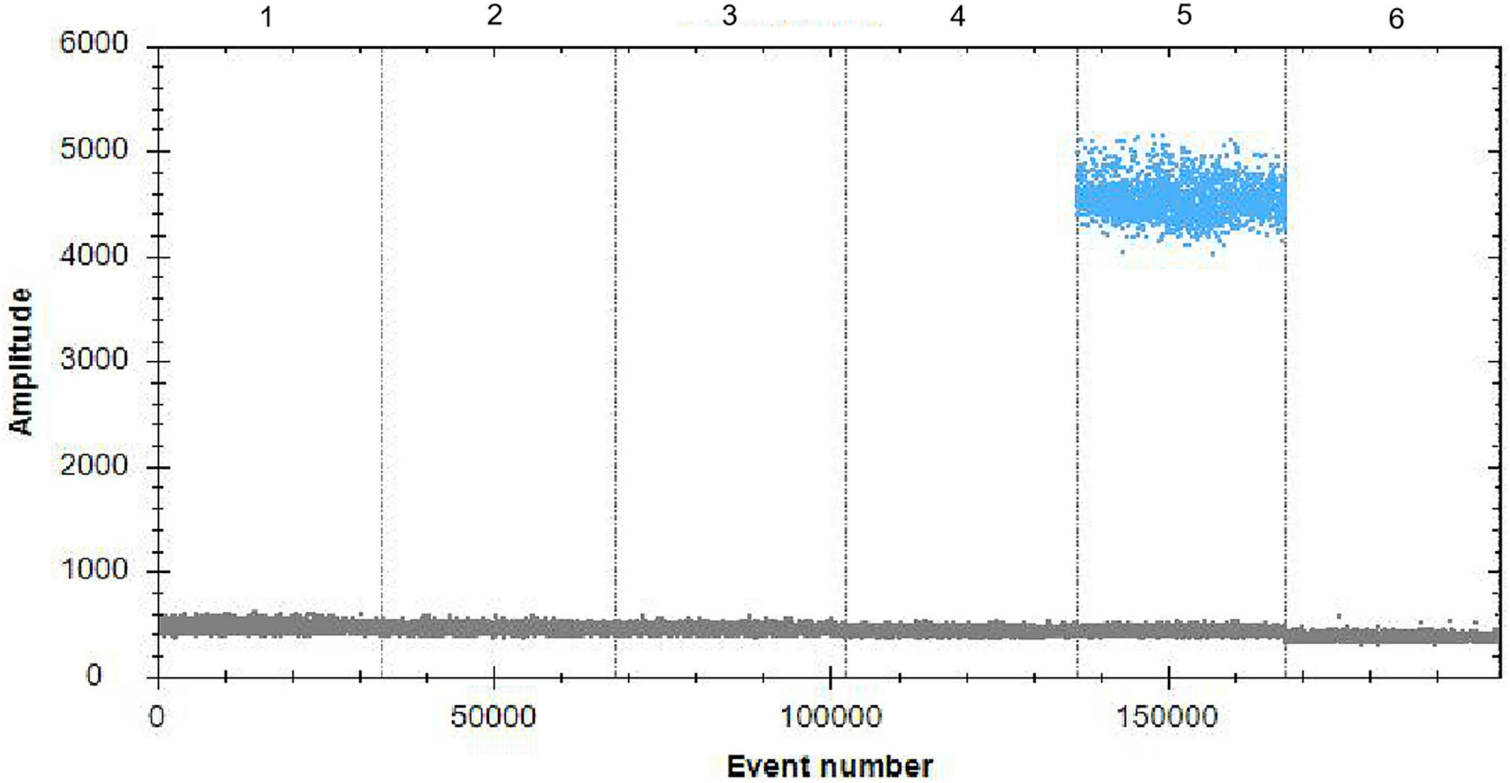
Specificity of ddPCR. 1–6: *Mycobacterium bovis*, *Escherichia coli* (O:157), *Salmonella* spp., *Streptococcus* spp., A19-ΔVirB12 *Brucella* vaccine, and *Clostridium perfringens*, respectively.

No copy numbers were detected with qPCR in addition to the DNA from A19-ΔVirB12 *Brucella* vaccine in the FAM channel (Figure 7), confirming that the primers and probes exhibited high specificity.

### 3.4 Detection of clinical samples

The commercial fluorescence quantitative reagent kits identified 32 positive samples with the percentage of *Brucella*-positive samples was 60.38%. In contrast, the ddPCR results showed 45 positive samples including two vaginal swabs positive for the A19-ΔVirB12 vaccines strain, and the percentage of *Brucella*-positive samples was 84.91% (Table 2). Thus, ddPCR exhibited higher sensitivity for positive samples compared with that of conventional qPCR and distinguish A19-ΔVirB12 vaccine strains and wild-type strains.

**Table 2:** Samples information in different cities.

City	No. of samples	Immune background of brucellosis	Sample types/number	Positive sample types/number detected by commercialized kits	Positive sample types/number detected by the ddPCR
Yantai	6	Not vaccinated	Milk/1; vaginal swabs/5	Milk/1; vaginal swabs/3	Milk/1; vaginal swabs/3
Linyi	6	Not vaccinated	Vaginal swabs/6	Vaginal swabs/4	Vaginal swabs/6
Qingdao	8	Not vaccinated	Milk/1; vaginal swabs/7	Milk/0; vaginal swabs/6	Milk/1; vaginal swabs/6
Weihai	10	Not vaccinated	Milk/2; vaginal swabs/8	Milk/2; vaginal swabs/5	Milk/2; vaginal swabs/8
Liaocheng	8	A19-ΔVirB12 vaccine	Milk/2; vaginal swabs/6	Milk/1; vaginal swabs/1	Milk/1; vaginal swabs/3 (one sample was positive for A19-ΔVirB12 vaccine)
Rizhao	5	A19-ΔVirB12 vaccine	Vaginal swabs/6	Vaginal swabs/3	Vaginal swabs/4 (two samples were positive for A19-ΔVirB12 vaccine)
Dongying	10	A19-ΔVirB12 vaccine	Milk/2; vaginal swabs/8	Milk/2; vaginal swabs/4	Milk/2; vaginal swabs/8 (6 samples were positive for A19-ΔVirB12 vaccine)

## 4 Discussion

ddPCR is a third-generation nucleic acid quantification technology. It involves separating the fluorescence-based PCR reaction mixture into tens of thousands of nanoliter-sized individual droplets. After PCR amplification, a droplet reader analyzes the fluorescence signal of each droplet, which determines the presence or absence of target nucleic acids (positive fluorescence indicates the presence of target). The fluorescence data are digitized, and analysis software is used to calculate the absolute copy number concentration of the target based on the Poisson distribution and the proportion of positive droplets. This allows for absolute quantification of low-abundance nucleic acid targets without reference standards or internal controls (Gerdes et al., 2016; Mehle and Dreo, 2019). ddPCR has been widely utilized to detect various infectious diseases, including *Mycobacterium tuberculosis* and HIV (Henrich et al., 2012).

Vaccination against *Brucella* remains the main strategy for brucellosis prevention and control. In China, the main vaccines available include *Brucella suis* S2, *Brucella melitensis* M5/M5-90, *Brucella abortus* A19, and the *Brucella abortus* A19-ΔVirB12 strains. The A19 vaccine strain, derived from the S19 strain, was introduced from the Soviet Union in the 1950s. It has been widely used for brucellosis prevention in dairy cattle since the 1930s and is recognized worldwide as the most effective vaccine for bovine brucellosis (Yang et al., 2013). The A19 strain is a smooth-type *Brucella* that induces a persistent antibody response after vaccination (Cheng et al., 2021). However, since both are specific antibodies, serological tests cannot distinguish vaccine-induced antibodies from those induced by natural infection. The A19-ΔVirB12 strain was developed in China by deleting the *VirB12* gene, part of the type IV secretion system in *Brucella*, from the A19 parent strain using homologous recombination technology. This mutant strain retain exhibits the same protective efficacy as the parental strain but exhibits reduced virulence and simultaneously carries a diagnostic marker (Yi et al., 2013).

The *Brucella VirB8* protein, an essential and highly conserved component of the type IV secretion system, functions as an early-stage secreted protein (Rouot et al., 2003; Patey et al., 2006). In this study, primers and probes were designed based on the *Brucella VirB8* fragment, suitable for genus-level identification. The absence of the *VirB12* sequence in the *Brucella abortus* A19-ΔVirB12 vaccine strain prevents its amplification. The optimal reaction system and conditions were determined, leading to the development of a duplex ddPCR assay for differentiating the A19-ΔVirB12 vaccine strain.

The single-digital PCR method for *Listeria bacteria* and SARS established by Witte et al. (2016) and Paolo et al. (2021), respectively, achieved a sensitivity of single copy per μL. Compared with the above-mentioned detection methods, the duplex ddPCR assay demonstrated detection sensitivities of 2.13 × 10^0^ copies/μL for *VirB8* and 2.26 × 10^0^ copies/μL for *VirB12*, attaining the detection sensitivity of single-digital PCR.

In the analysis of clinical samples, we found that the cattle from farms in four cities (Yantai, Linyi, Qingdao, and Weihai) had not been immunized with the *Brucella* vaccine. A positive result for *Brucella* nucleic acid indicated that the animal had been infected with *Brucella*. To prevent other animals and humans from being infected, the positive animals should be immediately subjected to harmless treatment. The results showed that the number of positive animals detected by ddPCR was significantly higher than that detected by commercial kits, which indicated that ddPCR was more effective in preventing the risk of the spread of *Brucella* infection in animals. As for cattle that had received the *Brucella abortus* A19-ΔVirB12 vaccine, the ddPCR assay was able to detect both the vaccine strain and wild-type *Brucella* infections. Consequently, at the cattle farms in the cities of Liaocheng, Rizhao and Dongying, cattle infected with wild-type *Brucella* were subjected to harmless disposal, while those immunized with the *Brucella abortus* A19-ΔVirB12 vaccine were exempted from such disposal-this measure effectively reduced the breeding costs for the farms. The test results showed that the ddPCR method had a higher detection rate and greater sensitivity compared with those of the commercial kits.

These findings suggest that clinical test samples for Brucellosis, such as milk samples and vaginal swabs, contain relatively low levels of bacteria, which requires higher sensitivity detection methods; therefore ddPCR is more suitable for such scenarios. Moreover, the ddPCR method established by us has greater advantages in detecting Brucellosis clinical samples with a *Brucella abortus* A19-ΔVirB12 vaccine background.

In conclusion, the duplex ddPCR assay established in this study exhibited excellent specificity and sensitivity. It facilitated accurate identification of *Brucella* in samples while effectively differentiating between strains from natural *Brucella* infections and immunization with the A19-ΔVirB12 vaccine strain, offering valuable technical support for preventing and controlling brucellosis. Meanwhile, the analysis of clinical samples revealed that *Brucella* infections existed in coastal cities of the Shandong Province, such as Qingdao, Yantai, and Weihai. Therefore, Brucellosis prevention and control efforts in these regions should be strengthened.

## Data Availability

The original contributions presented in the study are included in the article/supplementary material, further inquiries can be directed to the corresponding authors.
